# The distribution and spread of naturally occurring *Medea* selfish genetic elements in the United States

**DOI:** 10.1002/ece3.5876

**Published:** 2019-11-27

**Authors:** Sarah A. Cash, Marce D. Lorenzen, Fred Gould

**Affiliations:** ^1^ Program in Genetics Department of Biological Sciences North Carolina State University Raleigh NC USA; ^2^ Department of Entomology and Plant Pathology North Carolina State University Raleigh NC USA

**Keywords:** maternal effect, *Medea*, red flour beetle, selfish genetic element

## Abstract

Selfish genetic elements (SGEs) are DNA sequences that are transmitted to viable offspring in greater than Mendelian frequencies. *Medea* SGEs occur naturally in some populations of red flour beetle (*Tribolium castaneum*) and are expected to increase in frequency within populations and spread among populations. The large‐scale U.S. distributions of *Medea‐4* (M^4^) had been mapped based on samples from 1993 to 1995. We sampled beetles in 2011–2014 and show that the distribution of M^4^ in the United States is dynamic and has shifted southward. By using a genetic marker of *Medea‐1* (M^1^), we found five unique geographic clusters with high and low M^1^ frequencies in a pattern not predicted by microsatellite‐based analysis of population structure. Our results indicate the absence of rigid barriers to *Medea* spread in the United States, so assessment of what factors have limited its current distribution requires further investigation. There is great interest in using synthetic SGEs, including synthetic *Medea*, to alter or suppress pest populations, but there is concern about unpredicted spread of these SGEs and potential for populations to become resistant to them. The finding of patchy distributions of *Medea* elements suggests that released synthetic SGEs cannot always be expected to spread uniformly, especially in target species with limited dispersal.

## Graphical abstract

This figure compares the *Medea‐4* element distribution over time. The *Medea‐4* element in the United States is widespread, but no longer in a latitudinal distribution.

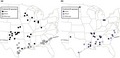

## 1 INTRODUCTION

Selfish genetic elements (SGEs) have been found to occur naturally in a huge variety of taxa, but despite decades of study by evolutionary biologists, the origins, mechanisms, and population‐level impacts of these elements are still largely unknown (Burt & Trivers, 2006). Many populations of red flour beetle (*Tribolium castaneum*) harbor naturally occurring selfish *Medea* elements (Beeman & Friesen, 1999). *Medea* elements are genomic sequences which cause death of the non‐*Medea* offspring of *Medea*‐bearing mothers (Beeman, Friesen, & Denell, 1992). Currently, the most parsimonious model suggests *Medea*'s action involves two tightly linked loci—one encoding a lethal, maternally expressed toxin in all eggs, and the other encoding a zygotic antidote that rescues only those progeny inheriting at least one *Medea* allele (Beeman & Friesen, 1999; Beeman et al., 1992). Because only non‐*Medea* (i.e., “wild‐type”) offspring produced by mothers that are heterozygous for the *Medea* element die, the *Medea* allele frequency is expected to increase within a population over time, provided that *Medea* introduction frequency is not extremely low, and the element does not carry a substantial fitness cost (Wade & Beeman, 1994). The influence of selfish genetic elements on populations and species can be substantial, from providing additional genetic variation that enables adaptation (e.g., Li, Schuler, & Berenbaum, 2007) to the lowering of population fitness (e.g., Carroll, Meagher, Morrision, Penn, & Potts, 2004). Beyond that, the basic population genetics and evolutionary history of elements such as *Medea* represent a fascinating yet understudied dimension in evolutionary biology (Burt & Trivers, 2006).

Beyond the importance of understanding natural SGEs for advancing basic science, knowledge of natural SGEs is relevant to the newly emerging technology of “gene drives” that aims at using synthetic SGEs to drive genes into pest populations that will act to suppress the populations or decrease vectorial capacity of the populations (e.g., Godfray, North, & Burt, 2017; Rode, Estoup, Bourguet, Courtier‐Orgogozo, & Debarre, 2019; Sinkins & Gould, 2006). Synthetic forms of *Medea* have been developed and tested in the laboratory (Buchman, Marshall, Ostrovski, Yang, & Akbari, 2018; Chen et al., 2007; Hay et al., 2010). Currently, there is concern among scientists and the public regarding predictability of spread of synthetic gene drives and the potential of pest populations to evolve resistance to the gene drive mechanisms or to linked sequences that impact traits and/or the viability of homozygote offspring (e.g., NASEM, 2016; Rode et al., 2019).

Two distinct *Medea* elements are known to be present in U.S. populations of red flour beetle: M^1^ and M^4^ (the M^2^ and M^3^ elements have each been identified only once, and in Asian populations) (Beeman & Friesen, 1999). Interestingly, M^1^ has only been found in wild beetles also harboring M^4^ (though the elements can be easily separated through crossing, and M^1^‐only strains are easily reared in the laboratory), while M^4^ is commonly found as the sole element present in wild populations (Beeman & Friesen, 1999). While both elements exhibit the same maternal‐effect lethality, they map to opposite ends of the same linkage group and do not cross‐rescue—for example, inheritance of an M^4^ allele cannot rescue the offspring of an M^1^‐bearing mother (Beeman & Friesen, 1999). M^1^ has been fully sequenced, and is associated with a transposon insertion, though the genetic mechanism involved in the maternal‐effect lethality remains a mystery (Lorenzen et al., 2008). A prior assessment of the distribution of the M^4^ element in the United States showed a striking latitudinal stepped cline (Beeman, 2003). All 26 sample locations above 33°N were fixed for the M^4^ element. In contrast, only two sampled locations below this latitude were fixed for the element, 21 lacked the element, and six had an intermediate frequency of M^4^. This delineation was so obvious that it spurred the hypothesis that distinct genetic races of *T. castaneum* might exist in the United States and that insufficient gene flow between these northern and southern races might create a barrier (Beeman, 2003). It is also possible that there is mating between the northern and southern populations, but that the southern populations are resistant to the action of *Medea*.

While they possess mechanisms allowing for their frequencies to increase, many naturally occurring SGEs appear to be maintained at low or intermediate frequency (reviewed in Hatcher, 2000). Despite their great potential for rapid spread, it is not uncommon for SGEs to be distributed in stable gradients. For example, the meiotic X‐chromosome driver *SR* in *Drosophila pseudoobscura* is distributed along a latitudinal gradient in North America, with the element more common in southern populations. This distribution appears to have been stable for at least the last half‐century potentially due to higher polyandry in northern populations (Price et al., 2014; Sturtevant & Dobzhansky, 1936). *Drosophila melanogaster* P elements show an east–west frequency gradient in Eurasia, with higher concentrations in western Europe fading out as sampling moves eastward (Anxolabéhère, Nouaud, Périquet, & Tchen, 1985). This gradient‐based distribution appears to be stable, likely due to the presence of “buffer populations” impervious to P elements (Bonnivard & Higuet, 1999).

Our goal was to determine whether the distribution of M^4^ in the United States is also stable, and if so, what genetic and/or environmental factors maintain the distribution. While M^1^ is also known to be present in U.S. populations, the only effort to assess its distribution involved few populations (Beeman & Friesen, 1999). Thus, it is unclear whether the same factors which shape the distribution of M^4^ also influence the distribution of M^1^.

*Tribolium castaneum* are thought to disperse primarily by human‐aided movement of infested stored grains and processed products. However, evidence for the importance of active dispersal via flight has been found in Australian populations of red flour beetle (Ridley et al., 2011). From traps spaced up to several dozen kilometers from the nearest food resources, roughly 88% of emigrating females were mated, showing the great potential for gene flow between populations. While the maximum extent of flight is unclear, it was evident that flight‐aided dispersal occurs at least on a scale of tens of kilometers (Ridley et al., 2011). Flight initiation peaks with warmer temperatures and increased daylight, so active dispersal will vary seasonally (Perez‐Mendoza, Campbell, & Throne, 2014).

Further, it is not yet known how successful long‐distance immigrants will be. *Tribolium castaneum* are not particularly attracted to undamaged or uninfested grains and flour, and success in locating a flour patch decreased as distance increased over a scale of only many centimeters (Romero, Campbell, Nechols, & With, 2010). The effectiveness of pheromones as attractants has been demonstrated on a scale of several meters (Boake & Wade, 1984; Obeng‐Ofori & Coaker, 1990), but may decay beyond this. If migrating beetles are effectively "flying blindly" until they luck upon a trail of aggregation pheromone or other food volatiles, the true impact of active dispersal on gene flow may be small.

An understanding of the factors which influence *Medea* spread is vital both for assessing *Medea*'s potential as a synthetic gene drive mechanism and for garnering a better understanding of the element's evolutionary biology. A critical first step is to describe current *Medea* distributions and determine whether the distributions are changing. Here, we describe our analysis of the contemporary distribution of M^4^ in the United States relative to the distribution found in the earlier survey. We also present a description of the large‐scale distribution of M^1^ in the United States. Finally, we address the potential role of population structure in shaping these distributions.

## 2 MATERIALS AND METHODS

### 2.1 Sample collection

Red flour beetles used to assess the current distribution of *Medea* elements M^1^ and M^4^ were collected between November 2011 and May 2014. Resampling of two sites originally sampled in 2012 occurred in September 2013 to determine whether there were frequency changes over time within populations. Collection sites included rice and wheat flour mills, feed mills, farm supply stores, grain elevators, grain bins, and other on‐farm grain storage. When appropriate, as determined by facility layout and researcher safety, pheromone‐baited traps or probes (Trécé, Inc.) were employed to collect beetles from mills or larger stores of grain. Otherwise, beetles were collected by hand or sifted from the substrate. In cases where visiting the sampling site was not possible, beetles were collected and shipped directly from the site to our laboratory by an extension agent or facility employee.

Dead beetles were frozen as soon as possible after collection. Live beetles were permitted to mate and oviposit for up to 3 weeks on a mixture of organic whole wheat pastry flour and 5% (by volume) brewer's yeast. These beetles were reared at 22–23°C and approximately 60% relative humidity in a controlled quarantine facility in order to establish cultures from each location; after this period, the originally collected beetles were removed and frozen for later genotyping. Cultures were maintained at the same temperature and humidity in a quarantine facility via periodic subculturing and flour replenishment.

In an effort to make inferences about an earlier distribution of M^1^, red flour beetles were also obtained from samples collected between 2004 and 2007. Samples from 2007 were collected in traps containing oil. This oil was removed from the beetles prior to DNA extraction by rinsing the beetles for 5 min in CitriSolv (Fisher Scientific), followed by a wash in double‐distilled water.

### 2.2 M^4^ diagnosis

Because we lacked a reliable M^4^ molecular marker at the time of these experiments, the presence of M^4^ in selected populations was assessed via crosses. Females from the homozygous M^4^ *pearl* strain were crossed to the non‐*Medea* GA‐1 strain (Haliscak & Beeman, 1983) to generate heterozygous M^4^ females. These females were crossed to males from wild populations, and after 3 days, eggs were counted for each cross. Once the offspring completed development, the final number of surviving adult progeny was tallied. To minimize the impact of potentially unhealthy females on the survivorship data, crosses producing fewer than 10 eggs, or those which failed to produce any surviving adults, were not included in the analysis. Offspring survival frequency was used as an indicator of the presence/absence of the M^4^ element in the wild‐derived males. As in Beeman (2003), survival means from each location were compared to wild type and M^4^ means using the Mann–Whitney test in MATLAB (Version 8.0.0.783; Mathworks).

The expectation for offspring from crosses between known M^4^‐heterozygous females and wild‐derived males of unknown M^4^ status is close to 100% survivorship if the male is actually homozygous for M^4^, roughly 75% for crosses to males heterozygous for M^4^, and about 50% offspring survivorship in crosses to males lacking the M^4^ element. We used this information to categorize the probable genotypes of individual beetles.

### 2.3 M^1^ genotyping

Because the M^1^ element has been fully sequenced, we were able to design M^1^‐specific primers and genotype via PCR.

Genomic DNA was extracted for 25 individual beetles per location (or, if 25 individuals were not available, as many individuals as possible) using the Qiagen DNeasy Blood and Tissue Kit. Primers used for amplification were as follows:

Forward primer: 5′‐TGGCGATAGTCAAAATCCTTTGTCG‐3′
M^1^ reverse: 5′‐TGCCACCTTCACGTAGCCCG‐3′
Wild‐type reverse: 5′‐CAGGGCCCCGGAGTATTTTTCC‐3′

PCRs were performed in 25 µl volumes, each containing 1× PCR buffer, 3.5 mM MgCl_2_, 200 µM dNTPs, 2 µl DNA template, 1 μmol forward primer, 0.5 μmol of each reverse primer, 0.5 U *Taq* DNA polymerase (Genesee), and ddH_2_O to 25 µl. Thermal cycling consisted of initial denaturation of DNA template at 95°C for 4 min followed by 40 cycles of 95°C for 30 s, 58°C for 45 s, and 72°C for 75 s, and a final extension step of 72°C for 5 min. Alleles were separated on 2.5% agarose gels infused with ethidium bromide and visualized by ultraviolet (UV) illumination.

### 2.4 Confirmation of M^1^ lethality

Because our assessment of the distribution of M^1^ relied on genotyping via a molecular marker, crosses were performed in select M^1^‐fixed populations to diagnose whether the M^1^ sequences amplified during genotyping represented fully functional M^1^ elements. Females from populations that were genotyped as fixed for M^1^ were crossed to males from the GA‐1 strain, a laboratory strain devoid of *Medea* elements. Five presumably M^1^‐heterozygous females derived from each cross were then paired with GA‐1 males, and eggs from each cross were counted, with the proportion of surviving offspring used to determine whether maternal‐effect lethality had occurred. Survival rates near 100% would indicate the absence of *Medea* elements, while survival rates near 50% would imply the presence of a *Medea* element in the source population. Five additional heterozygous females per population were backcrossed to males from their population of origin, and the proportion of surviving offspring (presumably 100%, as sires were expected to be homozygous M^1^) was used as a control.

To determine whether any maternal‐effect lethality uncovered in these populations could be attributed to the M^4^ element, three heterozygous females from each population were also crossed to males from the homozygous M^4^ *pearl* strain. The absence of rescue in these crosses indicated the presence of a functional *Medea* element other than M^4^, presumably M^1^, given that M^1^ is the only other *Medea* element that has been found in the United States.

### 2.5 Distribution analysis

We used SaTscan v.7 to identify any regional clustering in our observed M^1^ distribution (Kulldorff & Information Management Services, Inc., 2009). This allowed us to find patterns that were significantly different from a random distribution of genotype frequencies. The geographic coordinates of each sampling location were entered, along with the total number of individuals genotyped at that location and the number of M^1^ individuals identified. The program scans across the overall distribution in an elliptical window that was varied in radius from zero to a size exceeding the total sampling area, where each window represents a potential cluster of M^1^ or wild‐type genotypes (Table S4). For each window, a likelihood ratio was found by comparing the observed and expected number of M^1^ genotypes under a Bernoulli probability model. The window with the maximum likelihood was assigned a *p*‐value, obtained through Monte Carlo simulation (Kulldorff, 1997).

### 2.6 Microsatellite genotyping

We selected populations for analysis based on the number of individuals available from each sampled population, as well as the geographic location of each population. Sites in this study were selected that represented the wide geographic range of our sampling effort, while others were selected because of their proximity to other sampling locations, representing a finer scale. Populations with at least 20 sampled individuals were preferred. Selected primers described in Demuth et al. (2007) were used for microsatellite amplification (Table S2).

For cost‐effective, high‐throughput genotyping, forward primers were designed with a 5' M13 (−29) sequence CACGACGTTGTAAAACGAC, and a universal M13 (−29) primer labeled with either IRDye 800 or IRDye 700 was added to the reaction (Schuelke, 2000). The fluorescent IRDye is integrated on to the end of the fragment containing the forward primer, allowing for fragment detection and size estimation.

PCR was performed in 10 µl reactions, with each containing 2 µl genomic DNA (approximately 50 ng), 1× PCR buffer, 2 mM MgCl_2_, 0.2 mM dNTPs, 0.016 µM unlabeled forward primer, 0.06 µM reverse primer, 0.06 µM IRDye‐labeled universal M13 primer (Integrated DNA Technologies), 0.5 U *Taq* polymerase (Genesee), and ddH_2_O to 10 µl. The cycling conditions consisted of an initial denaturation step at 94°C for 4 min, followed by 15 cycles of 94°C for 30 s, 65°C (−1°C/cycle) for 30 s, and 72°C for 1 min and 20 s, and ending with 30 cycles of 94°C for 15 s, 50°C for 15 s, and 72°C for 45 s.

The PCR products were diluted with 10 µl ddH_2_O, and 10 µl of a formamide stop solution (95% formamide, 20 mm EDTA, bromophenol blue) was added, followed by a denaturing step consisting of 95°C for 5 min. Fragments were separated by electrophoresis on a 6.5% Long Ranger 1× TBE polyacrylamide gel, run on a Li‐Cor 4300 automated DNA sequencer at a constant power of 40 W at 45°C for 1.5 hr. Fragments were sized using a 50–350 bp IRDye 800 or 700 standard (Li‐Cor, Inc). Allele sizes were scored using QuantarPro software (KeyGene). Individuals missing data at more than three loci were removed from further analyses. Because this left population SC‐2 with only 10 individuals, it was excluded from population‐level analyses.

### 2.7 Genetic diversity and differentiation

Observed and expected heterozygosity and deviation from Hardy–Weinberg equilibrium were assessed in Arlequin (Excoffier et al., 2005). Genepop (Raymond & Rousset, 1995) was used to carry out exact tests for linkage disequilibrium, employing a Markov chain with 10,000 dememorization steps, 250 batches, and 2,500 iterations per batch to estimate the exact *p*‐value. Significance levels of the tests were adjusted for multiple comparison following standard Bonferroni corrections (Rice, 1989). Pairwise *F*_ST_ values were also estimated in Arlequin. Global *R*_ST_, allelic richness, and within‐population gene diversity were estimated in FSTAT (Goudet, 2001).

The program FreeNA (Chapuis & Estoup, 2007) was used to estimate null allele frequencies and to correct *F*_ST_ values for bias from null allele presence. A global M^1^ *F*_ST_ estimate was calculated using this same methodology, by assigning allele sizes to both the M^1^ and wild‐type alleles and constructing individual genotypes corresponding with our PCR genotyping results.

Potential population structure was investigated using the program STRUCTURE (Pritchard, Stephens, & Donnelly, 2000) to assess the most likely number of clusters (*K*), where both the "admixture" and "no admixture" models were run, with sampling location used as a prior, and allele frequencies correlated. Five replicates each from *K* = 1 to *K* = 15 were run with a burn‐in period of 200,000 steps followed by 200,000 MCMC iterations. The most likely value of *K* was determined in Structure Harvester (Earl & vonHoldt, 2012), which uses the delta *K* method described by Evanno, Regnaut, and Goudet (2005). For individual cluster assignments, STRUCTURE was run again for 20 replicates at the most likely *K*. The programs CLUMPP and DISTRUCT were used to visualize the raw data outputs from STRUCTURE (Jakobsson & Rosenberg, 2007).

The extent of genetic isolation due to geographic distance was assessed via a Mantel test with 10,000 permutations in Genepop's Isolde program (Raymond & Rousset, 1995), using two semimatrices: one consisting of *F*_ST_/(1−*F*_ST_) with ENA corrected *F*_ST_ values and another semimatrix of natural log‐transformed kilometer linear distances between sample locations. These analyses were also performed with the original uncorrected *F*_ST_ values, and the results obtained were compared to assess whether there were significant differences.

## 3 RESULTS

### 3.1 M^4^ genotyping and distribution

**Figure 1:**
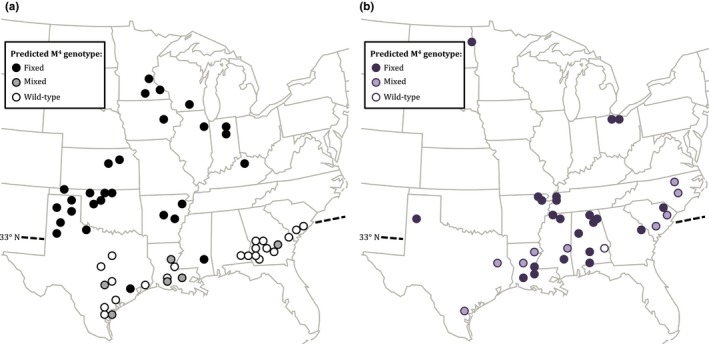
The M^4^ element in the United States is widespread, but no longer in a latitudinal distribution. (a) M^4^ distribution described by Beeman (2003), sampled 1993–1995. Figure adapted by authors from Beeman (2003) with permission. (b) M^4^ distribution of present study, sampled 2011–2014. Open circles indicate beetles genotyped were homozygous wild‐type, dark circles indicate beetles were homozygous M^4^, while light circles indicate both wild‐type and M^4^ beetles were present in the sample. The 33rd parallel (site of M^4^ delineation from Beeman, 2003) is indicated for reference

The overall result of testcrosses was that the M^4^ element was geographically widespread and found in nearly every sample population tested (Figure 1b). For most locations, all tested individuals were predicted to carry at least one M^4^ allele. This includes most locations south of 33°N, the previously described boundary of M^4^ fixation, indicating that M^4^ had moved southward. Only a single population, from southern Alabama, appeared to lack the M^4^ element. Two populations with intermediate predicted M^4^ frequencies were found in North Carolina, north of the 33rd parallel. North Carolina was not represented in the previous survey, and so it is unclear whether this represents a recent introduction of M^4^, or maintenance of an intermediate frequency.

Out of a total of 176 productive diagnostic crosses, survival above 100% was found in two—in each case, this was the result of counting exactly one more surviving adult than the number of eggs originally tallied. This was likely the result of an egg eluding the initial census by sticking to the side of the vial, or of an unfortunate miscount. These errors indicate that there may have been undercounts in some of the other replicates (Table S3).

### 3.2 M^1^ genotyping and distribution analysis

M^1^ diagnostic crosses confirmed that our PCR‐based M^1^ genotyping was indeed amplifying genomic regions representing functional M^1^ sequence. For each of the test populations that genotyped as fixed for M^1^ with our PCR assay, maternal‐effect lethality was apparent in crosses to non‐*Medea* individuals (Figure S1). Further, this lethality could not be rescued by the M^4^ element (Figure S1). We did not find any M^1^ beetles present in AL‐9, the only population sample to have lacked M^4^ in our analyses (Table S3).

**Figure 2:**
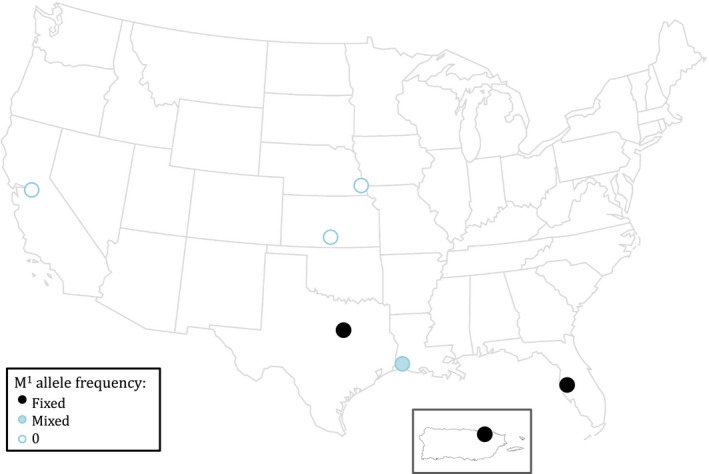
The M^1^ element was more prevalent in southern latitudes in wild beetles sampled 2004–2007. Open circles indicate beetles genotyped were homozygous wild‐type, dark circles indicate beetles were homozygous M^1^, while light circles indicate both wild‐type and M^1^ beetles were present in the sample. A sample from Puerto Rico is shown in an insert

While we did not obtain enough archived samples from 2004 to 2007 to make a full, comprehensive assessment of the prior distribution of M^1^ (Table S1), the data we collected still reflect heterogeneity in the M^1^ distribution (Figure 2). In contrast with the distribution of M^4^ described by Beeman (2003), where the M^4^ element was fixed in northern regions and largely absent at the southern extreme of the sampled region, we see M^1^ at high frequency in the southern portion of the sampled area (with the exception of the relatively low frequency observed in the Louisiana locality), and largely absent at higher latitudes.

**Figure 3:**
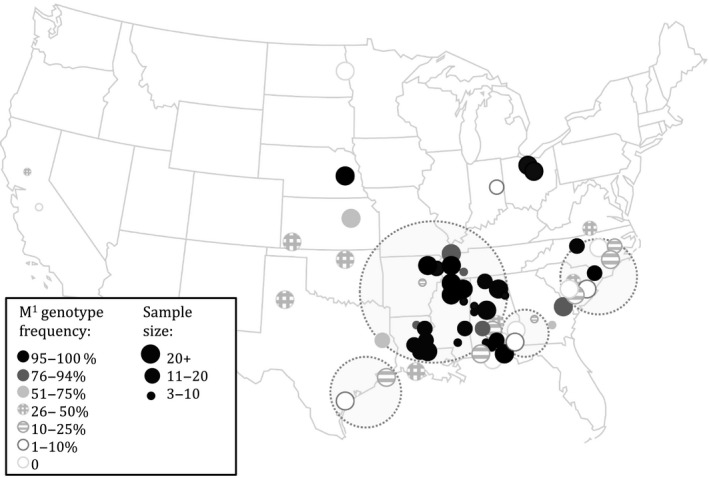
The present‐day distribution of the M^1^ element in the United States is patchy, with several significant clusters of high or low M^1^ frequency. Dotted circles indicate sites clustered by M^1^ allele frequency by SaTscan analysis. The color of the solid circle indicates the M^1^ genotype frequency of individuals sampled from that site, while the size of the circle is proportional to the number of individuals genotyped

The present‐day distribution of the M^1^ element in the United States (Figure 3) does not appear to show any latitudinal pattern, and certainly not one as distinct as the delineation found in prior M^4^ studies. There is, however, an interesting apparent clustering of high‐frequency *Medea* samples in the south‐central region, covering much of northern Alabama, northern Mississippi, western Tennessee, and eastern Arkansas.

Although the small number of sampled locations does not allow for a thorough comparison of the 2004–2007 distribution with the current‐day distribution, the pattern seen in the 2004–2007 samples is interesting, nonetheless. Our distribution analysis for the 2011–2014 samples revealed five nonoverlapping clusters (Figure 3). The largest cluster consisted of high‐frequency sites, incorporating 24 sampling locations stretching from Texas to eastern Alabama. Another high‐frequency cluster included three populations from Florida and southern Alabama. The remaining three clusters highlighted low‐frequency M^1^ regions: the eastern Carolinas (9 locations), eastern Alabama/western Georgia (4 locations), and the gulf coast (2 locations).

### 3.3 Genetic diversity and differentiation

The microsatellite markers were highly polymorphic, ranging between 6 and 31 alleles per locus across all samples (Table S5). Pairwise *F*_ST_ across all loci and locations ranged from a high of 0.264 (between NE and SC) to a low of 0.002 (between MS and SC). Global per‐locus *F*_ST_ values averaged 0.0669 (and ranged from 0.0365 to 0.1083, Table S7) and were comparable to another study of microsatellites in U.S. red flour beetle populations (0.018–0.149 in Semeao et al., 2012). In stark contrast, the *F*_ST_ for the M^1^ locus was 0.640, roughly six times higher than our most differentiated microsatellite locus.

When looking at microsatellite loci at all sample locations, we find a trend toward isolation by distance (IBD) (using corrected *F*_ST_, *r* ^2^ = .012, *p* = .03); however, when excluding food production sites, a positive correlation between genetic distance and geographic distance remains, but the correlation is no longer statistically significant (*p* = .094) (Figure S3). Driving this trend toward IBD, these sites tended to have higher average pairwise *F*_ST_ values than other locations (Table 1) and were also typically farther away from the next sampled location (the average pairwise geographic distance for these food production facilities was 1,094 km; the average pairwise distance *excluding* these sites was 861 km). Overall, samples from these sites also showed significantly lower allelic richness, lower observed heterozygosity, and lower within‐population genetic diversity. We tested to determine whether there was correlation between latitude and M^1^ frequency based on a sample of 31 populations. The correlation of 0.240 was not significant. We also tested for a correlation between M^1^ frequency and level of heterozygosity with the hypothesis that beetle populations with M^1^ would have lower heterozygosity due to linkage between the invading Medea element and alleles at other loci. We found no correlation (*r* = −.003).

**Table 1:** Beetle samples from food production facilities showed lower metrics of diversity than beetles sampled in other locations

	Food production	Other	*p*‐Value
Allelic richness	4.353	5.094	.004
*H*_O_	0.428	0.498	.027
*H*_S_	0.576	0.670	.001
*F*_IS_	0.258	0.257	.982
*F*_ST_	0.152	0.043	.001

Note

Shown are the average allelic richness, observed heterozygosity (*H*_O_), within‐population gene diversity (*H*_S_), inbreeding coefficient (*F*_IS_), and *F*_ST_ for the seven food production sites compared with the remaining 22 sampling locations. *p*‐Values were obtained after 1,000 permutations in FSTAT (Goudet, 2001).

No clear correlation was found between microsatellite and M^1^ frequency differences among populations (Figure S2b). We conclude from this that population structure does not appear to be a major factor shaping the *Medea* distributions. While the most likely number of clusters (*K*) from the structure analysis of microsatellite data was two, the delta *K* value (5.06) was quite small (Table S6). There was no clear geographic interpretation for the assignments, nor any clear relationship between clustering and *Medea* frequency.

## 4 DISCUSSION

Characterization of the spatial and temporal distribution of *Medea* elements is important for answering questions about *Medea* element evolution. Furthermore, from an applied perspective, there has been much attention paid to the potential use of *Medea* elements to spread beneficial genes into pest populations, but the only supporting data are from laboratory studies (Akbari et al., 2014; Buchman et al., 2018; Chen et al., 2007).

The current study is the first to assess the temporal dynamic nature of the distribution of *Medea* elements in a realistic landscape, and our data demonstrate that the M^4^ distribution has expanded in the past two decades (Beeman, 2003). Further, we present the first detailed descriptions of the distribution of M^1^ in the United States. We show that the M^1^ element is widespread but patchy, with some evidence of clusters of sampled areas with high and low frequencies. Finally, we show that there is little differentiation in frequencies of microsatellite alleles among the beetles collected in our geographically widespread samples, suggesting that *Medea* spread is not strongly restricted by gene flow. Other studies of red flour beetle, both in U.S. populations (Semeao et al., 2012) and in Australia (Ridley et al., 2011), have demonstrated similar levels of overall genetic differentiation among *T. castaneum* populations.

### 4.1 *Medea‐4* has spread geographically

Our M^4^ genotyping demonstrates recent spread of the M^4^ element within southern regions of the United States. This increase in M^4^ frequency in the southern United States indicates that there are no rigid boundaries—genetic, environmental, or dispersal—preventing the spread of M^4^ in the United States. The spread of M^4^ in the southern United States as well as our analysis of population structure does not support the existence of distinct geographic *T. castaneum* races that had been hypothesized earlier (Beeman, 2003). Assessment of M^4^ status of each individual beetle required crosses and assessment of offspring survival, so sample size was small, and we could only determine whether M^4^ was likely absent, present, or fixed in a population.

### 4.2 *Medea‐1* is widely but nonrandomly distributed within the United States

Because we have a molecular marker for active M^1^ elements, we were able to much more efficiently screen beetles for its presence/absence than was possible for M^4^, and we could roughly estimate frequency of M^1^ within populations. We were also able to check for the M^1^ marker in older, frozen beetles. Although M^1^ was present in most tested locations, the element was noticeably absent in some sampled regions, as revealed by our geographic clustering analysis. It is interesting to note that the lowest frequency clusters were in coastal regions. Because we lack a prior comprehensive, large‐scale M^1^ distribution for comparison, we do not yet know the temporal nature of these clusters.

Interestingly, M^1^ in our samples remains intertwined with M^4^. Even though the two elements can be easily separated in the laboratory with simple crosses, and with no apparent decrease in viability/fecundity of the M^1^‐only stocks, no M^1^ alleles were detected in beetles determined to lack the M^4^ genotype. One possible explanation is that M^1^ arose in an M^4^ background and has been tied to the M^4^ element's maternal‐effect lethality ever since. In the event of a dual introduction of M^1^ and M^4^ into a *Medea*‐naïve population (or an M^4^ population), we would expect both elements to persist, provided the introduction frequencies were not extremely low (Cash, 2016). Based on model predictions, in the case of a low‐frequency introduction, it is possible for just one of the two introduced *Medea* elements to be lost from the population based on recombination in heterozygous males.

### 4.3 Sampling locations and methodologies may impact differentiation metrics

A prior study of flour beetle populations in several U.S. commercial grain storage and processing facilities found evidence of population structure not associated with either geographic distance or the commodity type (rice or wheat) (Semeao et al., 2012). Beetle populations in these facilities are impacted by pest control treatments, resulting in population reduction or elimination. In a long‐term study of flour mills, the average number of beetles trapped postfumigation decreased by nearly 85% from prefumigation levels, indicating a significant population decrease (Campbell, Toews, Arthur, & Arbogast, 2010). Although these populations can rebound after treatment through propagation or immigration, the genetic makeup of the population is likely impacted by the bottleneck. In the present study, pairwise *F*_ST_ of two samples taken at the same facility in Ohio roughly 16 months apart was 0.099, higher than the average pairwise *F*_ST_ of 0.070 across all U.S. sites sampled. While pest management strategies are likely employed at other facilities, if storage or processing of products is not for human consumption, treatments may be less frequent or less stringent. An important caveat is that unlike most of the sampling locations, beetles from these sites were not collected directly by the researchers, who made an effort to collect from several locations within a site when possible. Instead, these beetles were sent by the facility, and may represent a nonrandom sample of the overall population, resulting in the lower levels of diversity seen.

### 4.4 Updating hypotheses about *Medea* history

While some other SGEs have stable regional distributions (Bonnivard & Higuet, 1999; Price, Hoskyns, Rapley, Evans, & Wedell, 2012), we have presented evidence for a dynamic *Medea* distribution in the United States. *Medea* distributions in other regions may be more stable, and could be influenced by ecological factors, or the presence of nonfunctional neutral *Medea* alleles or suppressors. Such regions could include the boundary between M^4^ and the H suppressor element in Asian populations (Thomson & Beeman, 1999; Thomson, Friesen, Denell, & Beeman, 1995), or low‐frequency regions such as Australia (Beeman & Friesen, 1999); however, the stability of these distributions has yet to be investigated.

The generation time of *T. castaneum* under optimal conditions is roughly 5 weeks at 30°C. However, development slows dramatically with temperature decreases and eggs fail to hatch below 17.5°C, halting reproduction in cooler months (Howe, 1956). With a *Medea* element that has no associated fitness cost, the time between immigration into a population at moderate frequency (10%) and fixation could take 2–5 years (Ward et al., 2010). If the initial frequency in a population is low (1%), fixation could take decades. Still, unless introductions to the United States were very recent, a *Medea* with no fitness cost would be expected to be spread more widely. Importation of *T. castaneum‐*infested grains and other stored commodities from regions where *Medea* is uncommon may reintroduce wild‐type beetles, suppressing the spread of *Medea* elements. There is a dramatic contrast between the lack of substantial microsatellite allelic differentiation found among our geographic populations and the strong differentiation in M^1^ allele frequencies among these same populations. If M^1^ had been in the United States for thousands of years, the clusters that we found would not be expected unless there was some physical, ecological, or genetic barrier to spread. In a companion study (Cash, Robert, Lorenzen, & Gould, in review), we tested to determine whether *Medea* spread is inhibited when it is introduced to laboratory colonies of beetles from populations that lack *Medea*.

While the current study reveals interesting information about the spatial and temporal dynamics of *Medea* elements, it is limited in scope. Future studies could examine distributions of *Medea* elements on a finer geographic scale and also could be expanded into other countries. Once there are molecular markers for multiple *Medea* elements, such studies would become less labor‐intensive.

Recent advances in molecular biology have enabled the development of synthetic *Medea* elements (Buchman et al., 2018; Chen et al., 2007; Hay et al., 2010) and other synthetic gene drive mechanisms (Macias, Ohm, & Rasgon, 2017) with the goal of suppressing pest populations or eliminating traits that cause their pest status (Gantz & Akbari, 2018; Piaggio et al., 2017). To date, these synthetic drives have only been tested in laboratory settings (e.g., Kyrou et al., 2018) and release in the field is complicated by the fact that the extent of spread and the potential for resistance to the drive is hard to predict (NASEM, 2016). A more detailed understanding of the temporal dynamics of *Medea* and other naturally occurring selfish genetic elements in species that differ in population structure could provide insights that would aid in the assessment and testing of synthetic gene drives. The finding in this study of patchy distributions of *Medea* elements suggests that releases of synthetic selfish genetic elements to alter pest population traits such as ability to transmit human diseases cannot be expected to spread uniformly, especially in target species such as *Aedes aegypti*, (the vector of dengue, Zika, yellow fever) that have limited dispersal.

## CONFLICT OF INTEREST

None declared.

## AUTHOR CONTRIBUTIONS

SAC, FG, and MDL designed the research; MDL provided reagents; SAC performed the research, analyzed the data, and wrote the original draft; SAC, FG, and MDL interpreted the data, and wrote, reviewed, and edited the manuscript.

## Data Availability

More data can be accessed in the following thesis: Cash, S. A. 2016. An Experimental and Theoretical Analysis of the Selfish Genetic Element Medea in Red Flour Beetle Populations. PhD thesis, North Carolina State University, Raleigh. Data have been archived on Dryad https://doi.org/10.5061/dryad.7sqv9s4p3.
