# Supplementary material for: The distribution and spread of naturally occurring Medea selfish genetic elements in the United States
Source: Ecol Evol. 2019 Nov 27;9(24):14407–16. doi: 10.1002/ece3.5876 (PMC6953677; doi:10.1002/ece3.5876)

**SUPPLEMENTARY MATERIAL**

**Table S1.** **Beetles used to assess a prior distribution of the *Medea-1* element.** The origins of these beetles, along with their collection dates and number genotyped per sample location, is shown.

| Sample origin | Collection date | N |
| --- | --- | --- |
| Kansas | June 2004 | 20 |
| Texas | June 2007 | 20 |
| Florida | July 2007 | 12 |
| Puerto Rico | July 2007 | 14 |
| California | August 2007 | 20 |
| Nebraska | August 2007 | 20 |
| Louisiana | September 2007 | 20 |

**Table S2.** **Primers used to amplify microsatellite loci**. Sequences are from Demuth *et al.* (2007), with an added 5' M13 (-29) region for high-throughput genotyping (underlined).

| **Locus** | **Forward** | **Reverse** |
| --- | --- | --- |
| Tca-X.2 | CACGACGTTGTAAAACGACTGTAAATGGGTTTCCTGTTGT | AAATTCATTGTCATCAATTCACTCA |
| Tca-3.2 | CACGACGTTGTAAAACGATATGTTTCCGGGTTTTGAGG | TTTCTCATACTTTTGCCGGG |
| Tca-4.7 | CACGACGTTGTAAAACGACAATATTGCGACGGCCCTAGT | CACACGTCAAATTCAGAACAAA |
| Tca-5.13 | CACGACGTTGTAAAACGACATGAACAACCCTCGCATCTC | GCGCAATTTATATGCCCATT |
| Tca-6.18 | CACGACGTTGTAAAACGAAGATCCCAATGGGCAAATCT | GCCGAAACTTTGGGTGATAA |
| Tca-8.6 | CACGACGTTGTAAAACGACTCCTGGACACAATCTCCCTAA | GCGTGGGTCGGATAGATATG |
| Tca-9.1 | CACGACGTTGTAAAACGAAGCCGCAACAAAGTAAGCAA | TTCTGACTACCACCGACAGATTT |
| Tca-9.24 | CACGACGTTGTAAAACGATCAACGATAATAAAACATGTCACG | GTGAGCAACAGTCTCGCAAA |

**Table S3.** **Genotyped M^1^ and predicted M^4^ genotype of tested sample sites reveal a widespread presence of *Medea* in the United States.** Sample origin, N: number of genotyped M^1^ individuals, OR M^4^ crosses performed for that sample location; average offspring survival in M^4^ tests, and predicted M^4^ status of laboratory and wild-derived individuals tested for the M^4^ element. Results of Mann-Whitney test comparisons to non-M^4^ (+/+) and M^4^ averages are shown. NA = not applicable

|  |  | **M^1^ Genotyping** | | | **M^4^ Genotyping** | | | | | |
| --- | --- | --- | --- | --- | --- | --- | --- | --- | --- | --- |
| **Sample Name** | **Sample Origin (County/Parish)** | **N** | **M^1^ Genotype Frequency** | **M^1^ Allele Frequency** | **N** | **% Survival of Each Cross** | **Avg. % Survival**  **(+/- sd)** | **+/+** | **M^4^/M^4^** | **Predicted M^4^ type** |
| GA-1 | - |  |  |  | 5 | 55  50  47  46  26 | 45 + 11.2 | NA | 0.0079 | + |
| *pearl* (M^4^) | - |  |  |  | 5 | 100  100  98  97  91 | 98 + 3.7 | 0.0079 | NA | M |
| AL-1 | Autauga | 10 | 0.3 | 0.3 |  |  |  |  |  |  |
| AL-2 | Geneva | 68 | 0.04 | 0.04 |  |  |  |  |  |  |
| AL-3 | Geneva | 4 | 0 | 0 |  |  |  |  |  |  |
| AL-4 | Conecuh | 63 | 0.16 | 0.09 |  |  |  |  |  |  |
| AL-5 | Baldwin | 43 | 0.16 | 0.12 |  |  |  |  |  |  |
| AL-6 | DeKalb | 27 | 1 | 0.91 | 5 | 100  100  100  90  89 | 96 + 5.9 | 0.0079 | 1 | M |
| AL-7 | Fayette | 6 | 1 | 1 |  |  |  |  |  |  |
| AL-8 | DeKalb | 10 | 1 | 0.95 | 5 | 100  100  100  97  93 | 98 + 3.1 | 0.0079 | 0.7619 | M |
| AL-9 | Henry | 26 | 0 | 0 | 10 | 80  74  71  61  58  49  47  36  33  30 | 54 + 17.7 | 0.3237 | 0.0007 | + |
| AL-10 | Lawrence | 12 | 1 | 0.96 | 5 | 100  93  91  81  67 | 86 + 12.8 | 0.0079 | 0.1667 | M |
| AL-11 | Fayette | 25 | 1 | 0.96 | 5 | 104  100  97  95  87 | 97 + 6.6 | 0.0079 | 0.8651 | M |
| AL-12 | Fayette | 7 | 1 | 1 |  |  |  |  |  |  |
| AL-13 | Escambia | 6 | 1 | 1 |  |  |  |  |  |  |
| AL-14 | Monroe | 16 | 0.88 | 0.78 | 5 | 100  96  88  87  57 | 85 + 17.1 | 0.0079 | 0.1984 | M |
| AL-15 | Escambia | 17 | 1 | 0.85 | 5 | 102  96  95  83  74 | 90 + 11.5 | 0.0079 | 0.2778 | M |
| AR-1 | Monroe | 5 | 0.2 | 0.2 |  |  |  |  |  |  |
| AR-2 | Craighead | 14 | 1 | 1 | 3 | 100  97  94 | 97 + 2.9 | 0.0357 | 0.8929 | M |
| AR-3 | Craighead | 25 | 1 | 0.96 | 5 | 100  100  100  94  76 | 94 + 8.1 | 0.0079 | 1 | M |
| CA-1 | Butte | 3 | 0.33 | 0.33 |  |  |  |  |  |  |
| CA-2 | San Joaquin | 6 | 0 | 0 |  |  |  |  |  |  |
| FL-1 | Escambia | 9 | 1 | 1 |  |  |  |  |  |  |
| FL-2 | Escambia | 20 | 1 | 1 |  |  |  |  |  |  |
| FL-3 | Escambia | 25 | 0 | 0 |  |  |  |  |  |  |
| Ga-1 | Tift | 9 | 0.67 | 0.56 |  |  |  |  |  |  |
| Ga-2 | Sumter | 5 | 0.2 | 0.1 |  |  |  |  |  |  |
| Ga-3 | Columbia | 26 | 1 | 0.88 | 5 | 95  87  83  73  71 | 82 + 10 | 0.0079 | 0.0159 | MIXED |
| IN-1 | Wayne | 13 | 0.08 | 0.04 |  |  |  |  |  |  |
| KS-1 | Dickinson | 24 | 0.75 | 0.65 |  |  |  |  |  |  |
| KS-2 | Morton | 43 | 0.49 | 0.4 |  |  |  |  |  |  |
| LA-1 | Richland | 6 | 0.83 | 0.75 |  |  |  |  |  |  |
| LA-2 | Richland | 16 | 1 | 0.94 |  |  |  |  |  |  |
| LA-4 | Acadia | 22 | 0.5 | 0.34 | 5 | 100  100  95  95  81 | 94 + 7.9 | 0.0079 | 0.6825 | M |
| LA-5 | Franklin | 18 | 1 | 0.72 | 3 | 80  75  69 | 75 + 5.1 | 0.0357 | 0.0357 | MIXED |
| LA-6 | Avoyelles | 20 | 1 | 0.95 | 4 | 100  100  81  68 | 87 + 15.5 | 0.0159 | 0.6825 | M |
| LA-7 | Rapides | 19 | 0.95 | 0.89 | 2 | 98  59 | 78 + 27.1 | 0.0952 | 0.4762 | MIXED |
| LA-8 | Avoyelles | 11 | 1 | 0.82 | 5 | 100  98  84  75  66 | 84 + 14.5 | 0.0079 | 0.2381 | M |
| MS-1 | Marshall | 22 | 1 | 0.95 | 5 | 100  100  98  95  72 | 93 + 11.9 | 0.0079 | 0.9048 | M |
| MS-2 | Pontotoc | 22 | 1 | 0.98 | 5 | 100  100  100  80  61 | 89 + 17.5 | 0.0079 | 1 | M |
| MS-3 | Pontotoc | 14 | 1 | 1 |  |  |  |  |  |  |
| MS-4 | Pontotoc | 25 | 1 | 0.94 |  |  |  |  |  |  |
| MS-5 | Forrest | 15 | 1 | 0.97 | 4 | 100  87  86  68 | 85 + 13.3 | 0.0159 | 0.1905 | M |
| MS-6 | Lauderdale | 5 | 1 | 1 | 5 | 95  94  91  87  86 | 90 + 4.2 | 0.0079 | 0.0397 | M |
| NC-1 | Wilson | 17 | 0.12 | 0.12 |  |  |  |  |  |  |
| NC-2 | Wake | 21 | 0 | 0 | 5 | 97  82  75  63  45 | 73 + 19.6 | 0.0952 | 0.0238 | MIXED |
| NC-3 | Forsyth | 11 | 1 | 0.9 |  |  |  |  |  |  |
| NC-4 | Johnston | 25 | 0.2 | 0.15 | 11 | 93  80  68  61  58  57  55  52  38  32  25 | 56 + 20 | 0.1516 | 0.00092 | MIXED |
| ND-1 | Grand Forks |  |  |  | 5 | 100  100  100  95  83 | 96 + 7.4 | 0.0079 | 1 | M |
| NE-1 | Lancaster | 22 | 1 | 1 |  |  |  |  |  |  |
| OH-1 | Seneca (2011) | 26 | 1 | 0.9 | 4 | 100  90  81  72 | 86 + 12 | 0.0159 | 0.1905 | M |
| OH-1B | Seneca (2013) | 25 | 1 | 0.96 | 5 | 100  100  100  100  94 | 99 + 2.6 | 0.0079 | 0.4048 | M |
| SC-1 | Dillon | 14 | 1 | 1 | 5 | 100  100  93  77  63 | 87 + 16.3 | 0.0079 | 0.4921 | M |
| SC-2 | Clarendon | 12 | 0.42 | 0.21 |  |  |  |  |  |  |
| SC-2B | Clarendon | 20 | 0.08 | 0.04 | 4 | 75  54  50  41 | 55 + 14.5 | 0.4603 | 0.0159 | + |
| SC-3 | Calhoun | 21 | 0.19 | 0.1 |  |  |  |  |  |  |
| SC-4 | Aiken | 18 | 0 | 0 | 10 | 78  74  58  57  57  54  53  53  48  41 | 57 + 10.9 | 0.0506 | 0.0007 | MIXED |
| TN-1 | Madison | 1 | 1 | 1 |  |  |  |  |  |  |
| TN-2 | Fayette | 10 | 0.9 | 0.85 |  |  |  |  |  |  |
| TN-3 | Obion | 24 | 0.79 | 0.67 | 5 | 100  100  100  96  94 | 98 + 2.7 | 0.0079 | 0.8413 | M |
| TN-4 | Dyer | 20 | 1 | 0.95 | 5 | 100  100  95  90  81 | 93 + 8.1 | 0.0079 | 0.4921 | M |
| TX-1 | Orange | 22 | 0.14 | 0.09 |  |  |  |  |  |  |
| TX-2 | Nueces | 95 | 0.04 | 0.03 | 6 | 97  74  63  52  51  50 | 64 + 18.2 | 0.0346 | 0.0130 | MIXED |
| TX-3 | Hale | 24 | 0.38 | 0.31 | 5 | 100  100  100  96  96 | 98 + 2.1 | 0.0079 | 0.8413 | M |
| TX-4 | Angelina | 17 | 0.65 | 0.47 | 5 | 100  79  79  67  67 | 78 + 13.6 | 0.0079 | 0.0794 | MIXED |
| VA-1 | Pittsylvania | 16 | 0.31 | 0.28 |  |  |  |  |  |  |

**Table S4. Clustering analysis revealed 17 clusters where M^1^ genotype frequency differed significantly from expectation.** Clusters are presented in order of decreasing significance. For each cluster is included: the names of sites included within that cluster, the numbers of any clusters overlapped, the radius of the cluster, the total population size within the cluster, the M^1^ genotype frequency as found by PCR genotyping, the expected genotype frequency (the frequency across the entire distribution), the and the log likelihood ratio and p-value.

| **Cluster**  **No.** | **Sites Included** | **Overlapping**  **Cluster Nos.** | **Cluster Radius (km)** | **Cluster Pop.** | **M^1^ Genotype**  **Frequency in Cluster** | **Expected M^1^ Genotype**  **Frequency** | **Log likelihood Ratio** | **p-value** |
| --- | --- | --- | --- | --- | --- | --- | --- | --- |
| 1 | AR-1, AR-3, AR-2, MS-1, TN-2, TN-4, MS-2, MS-3, MS-4, TN-3, LA-1, LA-2, LA-7, AL-7, AL-12, AL-11, MS-6, AL-10, LA-5, LA-6, LA-8, MS-5, AL-6, AL-8 | 3  6  7 | 492.38 | 378 | 0.968 | 0.544 | 236.895253 | < 1.0 x 10^-17^ |
| 2 | AL-9, AL-2, AL-3, Ga-2, AL-4, AL-1 | 5  13 | 162.31 | 176 | 0.097 | 0.544 | 90.779201 | < 1.0 x 10^-17^ |
| 3 | AL-10, AL-6, AL-11, AL-12, AL-7, AL-8, MS-4, MS-3 | 1 | 172.74 | 127 | 1 | 0.544 | 83.205914 | < 1.0 x 10^-17^ |
| 4 | TX-2, TX-1 | - | 412.87 | 117 | 0.060 | 0.544 | 69.734126 | < 1.0 x 10^-17^ |
| 5 | AL-9, AL-2, AL-3, Ga-2 | 2 | 132.07 | 103 | 0.039 | 0.544 | 67.918131 | < 1.0 x 10^-17^ |
| 6 | AR-1, AR-3, AR-2, MS-1, TN-2, TN-4, MS-2 | 1 | 209.74 | 117 | 0.957 | 0.544 | 55.532026 | < 1.0 x 10^-17^ |
| 7 | LA-1, LA-2, LA-7, LA-5, LA-6, LA-8 | 1 | 151.66 | 90 | 0.978 | 0.544 | 48.155633 | < 1.0 x 10^-17^ |
| 8 | NC-1, NC-4, NC-2, VA-1, SC-1, NC-3, SC-2, SC-2B, SC-3, SC-4 | 10  12 | 368.3 | 173 | 0.266 | 0.544 | 31.775138 | 7.2 x 10^-12^ |
| 9 | OH-1, OH-1B | - | 0 | 50 | 1 | 0.544 | 31.304036 | 1.1 x 10^-11^ |
| 10 | SC-2, SC-2B, SC-3, SC-4 | 8 | 107.14 | 75 | 0.147 | 0.544 | 27.168336 | 4.9 x 10^-10^ |
| 11 | AL-15, FL-2, AL-13 | - | 22.5 | 43 | 1 | 0.544 | 26.815303 | 6.8 x 10^-10^ |
| 12 | NC-1, NC-4, NC-2, VA-1 | 8 | 168.87 | 74 | 0.149 | 0.544 | 26.487712 | 9.2 x 10^-10^ |
| 13 | AL-4 | 2 | 0 | 63 | 0.159 | 0.544 | 21.110676 | 1.3 x 10^-7^ |
| 14 | FL-3 | - | 0 | 25 | 0 | 0.544 | 19.923653 | 3.8 x 10^-7^ |
| 15 | Ga-3 | - | 0 | 26 | 1 | 0.544 | 16.060873 | 1.3 x 10^-5^ |
| 16 | ND-1 | - | 0 | 20 | 0 | 0.544 | 15.890383 | 1.5 x 10^-5^ |
| 17 | NE-1 | - | 0 | 22 | 1 | 0.544 | 13.559990 | 1.3 x 10^-4^ |

**Table S5: Per-locus and per-population results of microsatellite analyses.** N: Number of individuals genotyped, N_A_: number of alleles per locus, N_PA_: number of private alleles per location, H_obs_: observed heterozygosity, H_exp_: expected heterozygosity, HWE: p-value (* p<0.05, **p<0.01 after Bonferroni correction).

| **Sample LocationName** | **Locus** | **N** | **N_A_** | **N_PA_** | **H_obs_** | **H_exp_** | **HWE** |
| --- | --- | --- | --- | --- | --- | --- | --- |
| All Sites | X.2  3.2  4.7  5.13  6.18  8.6  9.1  9.24 | \| 648 \| \| --- \| \| 648 \| \| 661 \| \| 677 \| \| 643 \| \| 673 \| \| 562 \| \| 642 \| | 31  24  24  7  6  9  16  7 |  | \| 0.683 \| \| --- \| \| 0.340 \| \| 0.372 \| \| 0.570 \| \| 0.683 \| \| 0.512 \| \| 0.170 \| \| 0.497 \| | \| 0.914 \| \| --- \| \| 0.798 \| \| 0.860 \| \| 0.523 \| \| 0.675 \| \| 0.610 \| \| 0.526 \| \| 0.630 \| | \| ** \| \| --- \| \| ** \| \| ** \| \|  \| \|  \| \| ** \| \| ** \| \| ** \| |
| AL-5 | X.2  3.2  4.7  5.13  6.18  8.6  9.1  9.24 | \| 20 \| \| --- \| \| 20 \| \| 20 \| \| 20 \| \| 20 \| \| 20 \| \| 13 \| \| 20 \| | 14  6  11  3  4  5  3  4 | 1 | \| 0.850 \| \| --- \| \| 0.200 \| \| 0.550 \| \| 0.500 \| \| 0.800 \| \| 0.350 \| \| 0.308 \| \| 0.800 \| | \| 0.921 \| \| --- \| \| 0.636 \| \| 0.885 \| \| 0.518 \| \| 0.699 \| \| 0.637 \| \| 0.446 \| \| 0.609 \| | \|  \| \| --- \| \| ** \| \| ** \| \|  \| \|  \| \|  \| \|  \| \| * \| |
| AL-6 | X.2  3.2  4.7  5.13  6.18  8.6  9.1  9.24 | \| 24 \| \| --- \| \| 24 \| \| 25 \| \| 25 \| \| 22 \| \| 25 \| \| 20 \| \| 22 \| | 15  7  9  4  5  4  4  4 |  | \| 0.750 \| \| --- \| \| 0.250 \| \| 0.160 \| \| 0.600 \| \| 0.773 \| \| 0.360 \| \| 0.100 \| \| 0.409 \| | \| 0.933 \| \| --- \| \| 0.777 \| \| 0.803 \| \| 0.549 \| \| 0.707 \| \| 0.615 \| \| 0.468 \| \| 0.580 \| | \|  \| \| --- \| \| ** \| \| ** \| \| * \| \|  \| \|  \| \| ** \| \|  \| |
| AL-9 | X.2  3.2  4.7  5.13  6.18  8.6  9.1  9.24 | \| 27 \| \| --- \| \| 28 \| \| 28 \| \| 27 \| \| 27 \| \| 28 \| \| 21 \| \| 28 \| | 12  8  11  4  4  4  5  4 |  | \| 0.852 \| \| --- \| \| 0.393 \| \| 0.500 \| \| 0.259 \| \| 0.667 \| \| 0.464 \| \| 0.333 \| \| 0.429 \| | \| 0.909 \| \| --- \| \| 0.681 \| \| 0.772 \| \| 0.407 \| \| 0.678 \| \| 0.616 \| \| 0.646 \| \| 0.642 \| | \|  \| \| --- \| \| ** \| \| ** \| \|  \| \|  \| \|  \| \| * \| \|  \| |
| AL-11 | X.2  3.2  4.7  5.13  6.18  8.6  9.1  9.24 | \| 25 \| \| --- \| \| 23 \| \| 25 \| \| 25 \| \| 25 \| \| 25 \| \| 25 \| \| 25 \| | 13  7  10  3  4  4  10  5 |  | \| 0.640 \| \| --- \| \| 0.130 \| \| 0.240 \| \| 0.520 \| \| 0.680 \| \| 0.640 \| \| 0.320 \| \| 0.440 \| | \| 0.913 \| \| --- \| \| 0.706 \| \| 0.862 \| \| 0.590 \| \| 0.548 \| \| 0.581 \| \| 0.778 \| \| 0.630 \| | \| ** \| \| --- \| \| ** \| \| ** \| \|  \| \|  \| \|  \| \| ** \| \|  \| |
| AR-3 | X.2  3.2  4.7  5.13  6.18  8.6  9.1  9.24 | \| 23 \| \| --- \| \| 23 \| \| 24 \| \| 24 \| \| 24 \| \| 24 \| \| 23 \| \| 24 \| | 11  6  9  3  4  6  6  4 |  | \| 0.870 \| \| --- \| \| 0.304 \| \| 0.167 \| \| 0.458 \| \| 0.667 \| \| 0.500 \| \| 0.304 \| \| 0.542 \| | \| 0.904 \| \| --- \| \| 0.795 \| \| 0.621 \| \| 0.407 \| \| 0.560 \| \| 0.582 \| \| 0.552 \| \| 0.595 \| | \|  \| \| --- \| \| ** \| \| ** \| \|  \| \|  \| \|  \| \| * \| \|  \| |
| FL-2 | X.2  3.2  4.7  5.13  6.18  8.6  9.1  9.24 | \| 18 \| \| --- \| \| 18 \| \| 19 \| \| 19 \| \| 18 \| \| 19 \| \| 15 \| \| 19 \| | 11  7  10  3  4  4  2  3 |  | \| 0.833 \| \| --- \| \| 0.333 \| \| 0.263 \| \| 0.684 \| \| 0.778 \| \| 0.526 \| \| 0.000 \| \| 0.421 \| | \| 0.863 \| \| --- \| \| 0.606 \| \| 0.788 \| \| 0.568 \| \| 0.633 \| \| 0.613 \| \| 0.331 \| \| 0.457 \| | \|  \| \| --- \| \| * \| \| ** \| \|  \| \|  \| \|  \| \| ** \| \|  \| |
| FL-3 | X.2  3.2  4.7  5.13  6.18  8.6  9.1  9.24 | \| 25 \| \| --- \| \| 22 \| \| 25 \| \| 26 \| \| 24 \| \| 25 \| \| 25 \| \| 26 \| | 14  7  10  3  3  3  6  3 |  | \| 0.720 \| \| --- \| \| 0.091 \| \| 0.240 \| \| 0.654 \| \| 0.583 \| \| 0.320 \| \| 0.040 \| \| 0.462 \| | \| 0.904 \| \| --- \| \| 0.588 \| \| 0.743 \| \| 0.539 \| \| 0.577 \| \| 0.381 \| \| 0.572 \| \| 0.555 \| | \| * \| \| --- \| \| ** \| \| ** \| \|  \| \|  \| \|  \| \| ** \| \|  \| |
| Ga-3 | X.2  3.2  4.7  5.13  6.18  8.6  9.1  9.24 | \| 25 \| \| --- \| \| 24 \| \| 24 \| \| 25 \| \| 25 \| \| 25 \| \| 21 \| \| 21 \| | 13  9  10  4  4  3  2  4 |  | \| 0.840 \| \| --- \| \| 0.375 \| \| 0.292 \| \| 0.440 \| \| 0.720 \| \| 0.560 \| \| 0.095 \| \| 0.476 \| | \| 0.903 \| \| --- \| \| 0.730 \| \| 0.684 \| \| 0.389 \| \| 0.709 \| \| 0.536 \| \| 0.251 \| \| 0.605 \| | \|  \| \| --- \| \| ** \| \| ** \| \|  \| \|  \| \|  \| \|  \| \|  \| |
| KS-1 | X.2  3.2  4.7  5.13  6.18  8.6  9.1  9.24 | \| 21 \| \| --- \| \| 22 \| \| 23 \| \| 22 \| \| 19 \| \| 24 \| \| 14 \| \| 21 \| | 12  5  9  4  6  3  2  4 |  | \| 0.524 \| \| --- \| \| 0.273 \| \| 0.261 \| \| 0.500 \| \| 0.684 \| \| 0.500 \| \| 0.000 \| \| 0.810 \| | \| 0.922 \| \| --- \| \| 0.655 \| \| 0.847 \| \| 0.443 \| \| 0.723 \| \| 0.487 \| \| 0.423 \| \| 0.663 \| | \| ** \| \| --- \| \| ** \| \| ** \| \|  \| \|  \| \|  \| \| ** \| \|  \| |
| KS-2 | X.2  3.2  4.7  5.13  6.18  8.6  9.1  9.24 | \| 26 \| \| --- \| \| 26 \| \| 26 \| \| 26 \| \| 25 \| \| 26 \| \| 23 \| \| 26 \| | 15  10  7  4  4  8  4  4 | 1 | \| 0.731 \| \| --- \| \| 0.192 \| \| 0.308 \| \| 0.654 \| \| 0.720 \| \| 0.615 \| \| 0.174 \| \| 0.192 \| | \| 0.885 \| \| --- \| \| 0.777 \| \| 0.757 \| \| 0.587 \| \| 0.667 \| \| 0.723 \| \| 0.467 \| \| 0.417 \| | \| * \| \| --- \| \| ** \| \| ** \| \|  \| \|  \| \|  \| \| ** \| \| ** \| |
| LA-4 | X.2  3.2  4.7  5.13  6.18  8.6  9.1  9.24 | \| 23 \| \| --- \| \| 24 \| \| 24 \| \| 24 \| \| 23 \| \| 24 \| \| 22 \| \| 24 \| | 13  7  7  4  4  7  5  4 |  | \| 0.652 \| \| --- \| \| 0.167 \| \| 0.333 \| \| 0.417 \| \| 0.826 \| \| 0.458 \| \| 0.364 \| \| 0.500 \| | \| 0.836 \| \| --- \| \| 0.696 \| \| 0.699 \| \| 0.363 \| \| 0.724 \| \| 0.674 \| \| 0.521 \| \| 0.637 \| | \|  \| \| --- \| \| ** \| \| ** \| \|  \| \|  \| \| * \| \|  \| \|  \| |
| MS-1 | X.2  3.2  4.7  5.13  6.18  8.6  9.1  9.24 | \| 20 \| \| --- \| \| 19 \| \| 21 \| \| 22 \| \| 22 \| \| 22 \| \| 19 \| \| 20 \| | 15  8  10  3  5  5  3  4 | 1 | \| 0.800 \| \| --- \| \| 0.421 \| \| 0.381 \| \| 0.455 \| \| 0.636 \| \| 0.500 \| \| 0.053 \| \| 0.400 \| | \| 0.922 \| \| --- \| \| 0.808 \| \| 0.841 \| \| 0.469 \| \| 0.698 \| \| 0.599 \| \| 0.397 \| \| 0.637 \| | \|  \| \| --- \| \|  \| \| ** \| \|  \| \|  \| \|  \| \| ** \| \| * \| |
| MS-2 | X.2  3.2  4.7  5.13  6.18  8.6  9.1  9.24 | \| 24 \| \| --- \| \| 25 \| \| 23 \| \| 25 \| \| 24 \| \| 25 \| \| 23 \| \| 25 \| | 12  8  11  4  3  4  4  3 | 1 | \| 0.500 \| \| --- \| \| 0.320 \| \| 0.391 \| \| 0.640 \| \| 0.708 \| \| 0.480 \| \| 0.087 \| \| 0.480 \| | \| 0.887 \| \| --- \| \| 0.756 \| \| 0.721 \| \| 0.607 \| \| 0.664 \| \| 0.600 \| \| 0.521 \| \| 0.605 \| | \| ** \| \| --- \| \| ** \| \| ** \| \|  \| \|  \| \|  \| \| ** \| \|  \| |
| MS-4 | X.2  3.2  4.7  5.13  6.18  8.6  9.1  9.24 | \| 27 \| \| --- \| \| 26 \| \| 27 \| \| 26 \| \| 27 \| \| 27 \| \| 26 \| \| 26 \| | 13  4  9  5  6  6  5  4 | 1 | \| 0.815 \| \| --- \| \| 0.269 \| \| 0.370 \| \| 0.808 \| \| 0.889 \| \| 0.778 \| \| 0.346 \| \| 0.462 \| | \| 0.904 \| \| --- \| \| 0.461 \| \| 0.829 \| \| 0.701 \| \| 0.706 \| \| 0.700 \| \| 0.598 \| \| 0.506 \| | \|  \| \| --- \| \| * \| \| ** \| \|  \| \|  \| \| * \| \| * \| \|  \| |
| NC-1 | X.2  3.2  4.7  5.13  6.18  8.6  9.1  9.24 | \| 19 \| \| --- \| \| 18 \| \| 18 \| \| 19 \| \| 18 \| \| 18 \| \| 15 \| \| 19 \| | 7  7  10  3  6  3  4  4 |  | \| 0.684 \| \| --- \| \| 0.500 \| \| 0.500 \| \| 0.842 \| \| 0.722 \| \| 0.222 \| \| 0.533 \| \| 0.579 \| | \| 0.799 \| \| --- \| \| 0.730 \| \| 0.876 \| \| 0.671 \| \| 0.727 \| \| 0.210 \| \| 0.572 \| \| 0.661 \| | \|  \| \| --- \| \|  \| \| ** \| \|  \| \|  \| \|  \| \| ** \| \|  \| |
| NC-2 | X.2  3.2  4.7  5.13  6.18  8.6  9.1  9.24 | \| 21 \| \| --- \| \| 23 \| \| 23 \| \| 24 \| \| 23 \| \| 24 \| \| 22 \| \| 23 \| | 9  5  8  3  5  3  2  2 |  | \| 0.857 \| \| --- \| \| 0.043 \| \| 0.565 \| \| 0.917 \| \| 0.609 \| \| 0.542 \| \| 0.000 \| \| 0.565 \| | \| 0.787 \| \| --- \| \| 0.600 \| \| 0.791 \| \| 0.635 \| \| 0.692 \| \| 0.435 \| \| 0.089 \| \| 0.414 \| | \|  \| \| --- \| \| ** \| \| * \| \| ** \| \|  \| \|  \| \|  \| \|  \| |
| NC-4 | X.2  3.2  4.7  5.13  6.18  8.6  9.1  9.24 | \| 16 \| \| --- \| \| 17 \| \| 15 \| \| 19 \| \| 10 \| \| 19 \| \| 14 \| \| 13 \| | 10  12  5  3  4  3  3  2 |  | \| 0.813 \| \| --- \| \| 0.647 \| \| 0.333 \| \| 0.526 \| \| 0.400 \| \| 0.421 \| \| 0.000 \| \| 0.462 \| | \| 0.901 \| \| --- \| \| 0.904 \| \| 0.772 \| \| 0.570 \| \| 0.642 \| \| 0.494 \| \| 0.349 \| \| 0.440 \| | \|  \| \| --- \| \| * \| \| ** \| \|  \| \|  \| \|  \| \|  \| \|  \| |
| ND-1 | X.2  3.2  4.7  5.13  6.18  8.6  9.1  9.24 | \| 17 \| \| --- \| \| 18 \| \| 17 \| \| 18 \| \| 18 \| \| 18 \| \| 16 \| \| 18 \| | 10  5  8  4  4  7  5  4 |  | \| 0.647 \| \| --- \| \| 0.278 \| \| 0.412 \| \| 0.389 \| \| 0.778 \| \| 0.611 \| \| 0.375 \| \| 0.167 \| | \| 0.806 \| \| --- \| \| 0.646 \| \| 0.766 \| \| 0.424 \| \| 0.605 \| \| 0.735 \| \| 0.623 \| \| 0.211 \| | \|  \| \| --- \| \| ** \| \| * \| \|  \| \|  \| \|  \| \| * \| \|  \| |
| NE-1 | X.2  3.2  4.7  5.13  6.18  8.6  9.1  9.24 | \| 24 \| \| --- \| \| 22 \| \| 22 \| \| 24 \| \| 24 \| \| 24 \| \| 23 \| \| 24 \| | 6  10  5  3  2  4  5  2 |  | \| 0.708 \| \| --- \| \| 0.545 \| \| 0.091 \| \| 0.708 \| \| 0.583 \| \| 0.458 \| \| 0.087 \| \| 0.292 \| | \| 0.722 \| \| --- \| \| 0.781 \| \| 0.216 \| \| 0.598 \| \| 0.422 \| \| 0.438 \| \| 0.700 \| \| 0.311 \| | \|  \| \| --- \| \| * \| \| * \| \|  \| \|  \| \|  \| \| ** \| \|  \| |
| OH-1 | X.2  3.2  4.7  5.13  6.18  8.6  9.1  9.24 | \| 26 \| \| --- \| \| 24 \| \| 26 \| \| 26 \| \| 24 \| \| 25 \| \| 22 \| \| 25 \| | 11  8  12  4  3  5  4  3 |  | \| 0.615 \| \| --- \| \| 0.500 \| \| 0.423 \| \| 0.346 \| \| 0.333 \| \| 0.200 \| \| 0.045 \| \| 0.400 \| | \| 0.883 \| \| --- \| \| 0.719 \| \| 0.885 \| \| 0.334 \| \| 0.415 \| \| 0.602 \| \| 0.289 \| \| 0.528 \| | \| ** \| \| --- \| \|  \| \| ** \| \|  \| \|  \| \| ** \| \| ** \| \|  \| |
| OH-1B | X.2  3.2  4.7  5.13  6.18  8.6  9.1  9.24 | \| 17 \| \| --- \| \| 21 \| \| 19 \| \| 21 \| \| 21 \| \| 21 \| \| 20 \| \| 20 \| | 6  5  6  2  4  3  2  3 |  | \| 0.353 \| \| --- \| \| 0.238 \| \| 0.316 \| \| 0.048 \| \| 0.238 \| \| 0.619 \| \| 0.050 \| \| 0.400 \| | \| 0.766 \| \| --- \| \| 0.719 \| \| 0.780 \| \| 0.048 \| \| 0.224 \| \| 0.487 \| \| 0.050 \| \| 0.651 \| | \| ** \| \| --- \| \| ** \| \| ** \| \|  \| \|  \| \|  \| \|  \| \| ** \| |
| OK-1 | X.2  3.2  4.7  5.13  6.18  8.6  9.1  9.24 | \| 23 \| \| --- \| \| 23 \| \| 26 \| \| 26 \| \| 25 \| \| 25 \| \| 21 \| \| 25 \| | 14  11  12  4  4  4  4  4 |  | \| 0.652 \| \| --- \| \| 0.478 \| \| 0.423 \| \| 0.615 \| \| 0.960 \| \| 0.480 \| \| 0.095 \| \| 0.560 \| | \| 0.933 \| \| --- \| \| 0.839 \| \| 0.903 \| \| 0.649 \| \| 0.718 \| \| 0.500 \| \| 0.331 \| \| 0.682 \| | \| ** \| \| --- \| \| ** \| \| ** \| \|  \| \| * \| \|  \| \| ** \| \|  \| |
| SC-2B | X.2  3.2  4.7  5.13  6.18  8.6  9.1  9.24 | \| 19 \| \| --- \| \| 25 \| \| 24 \| \| 24 \| \| 24 \| \| 22 \| \| 21 \| \| 25 \| | 13  7  11  4  4  4  3  5 |  | \| 0.526 \| \| --- \| \| 0.320 \| \| 0.208 \| \| 0.625 \| \| 0.833 \| \| 0.455 \| \| 0.238 \| \| 0.360 \| | \| 0.919 \| \| --- \| \| 0.679 \| \| 0.809 \| \| 0.547 \| \| 0.691 \| \| 0.554 \| \| 0.501 \| \| 0.601 \| | \| ** \| \| --- \| \| ** \| \| ** \| \|  \| \|  \| \|  \| \| * \| \| * \| |
| SC-3 | X.2  3.2  4.7  5.13  6.18  8.6  9.1  9.24 | \| 23 \| \| --- \| \| 22 \| \| 23 \| \| 25 \| \| 23 \| \| 25 \| \| 16 \| \| 18 \| | 14  13  9  4  5  5  3  3 |  | \| 0.783 \| \| --- \| \| 0.591 \| \| 0.609 \| \| 0.720 \| \| 0.609 \| \| 0.880 \| \| 0.063 \| \| 0.611 \| | \| 0.896 \| \| --- \| \| 0.887 \| \| 0.787 \| \| 0.631 \| \| 0.724 \| \| 0.718 \| \| 0.542 \| \| 0.608 \| | \|  \| \| --- \| \| ** \| \|  \| \|  \| \|  \| \|  \| \| ** \| \|  \| |
| TN-3 | X.2  3.2  4.7  5.13  6.18  8.6  9.1  9.24 | \| 25 \| \| --- \| \| 23 \| \| 25 \| \| 25 \| \| 25 \| \| 25 \| \| 19 \| \| 25 \| | 12  7  12  4  4  5  5  4 | 1 | \| 0.760 \| \| --- \| \| 0.522 \| \| 0.480 \| \| 0.640 \| \| 0.520 \| \| 0.560 \| \| 0.211 \| \| 0.440 \| | \| 0.868 \| \| --- \| \| 0.786 \| \| 0.883 \| \| 0.660 \| \| 0.641 \| \| 0.624 \| \| 0.521 \| \| 0.594 \| | \|  \| \| --- \| \| ** \| \| ** \| \|  \| \|  \| \|  \| \| ** \| \|  \| |
| TN-4 | X.2  3.2  4.7  5.13  6.18  8.6  9.1  9.24 | \| 20 \| \| --- \| \| 19 \| \| 19 \| \| 20 \| \| 20 \| \| 20 \| \| 11 \| \| 20 \| | 11  6  9  5  3  4  2  4 | 1  1 | \| 0.750 \| \| --- \| \| 0.105 \| \| 0.316 \| \| 0.800 \| \| 0.600 \| \| 0.250 \| \| 0.273 \| \| 0.700 \| | \| 0.894 \| \| --- \| \| 0.735 \| \| 0.878 \| \| 0.691 \| \| 0.636 \| \| 0.515 \| \| 0.368 \| \| 0.553 \| | \|  \| \| --- \| \| ** \| \| ** \| \|  \| \|  \| \| * \| \|  \| \|  \| |
| TX-2 | X.2  3.2  4.7  5.13  6.18  8.6  9.1  9.24 | \| 21 \| \| --- \| \| 21 \| \| 21 \| \| 21 \| \| 14 \| \| 21 \| \| 10 \| \| 11 \| | 11  8  12  4  5  5  2  3 |  | \| 0.524 \| \| --- \| \| 0.524 \| \| 0.667 \| \| 0.619 \| \| 0.786 \| \| 0.810 \| \| 0.000 \| \| 0.818 \| | \| 0.866 \| \| --- \| \| 0.847 \| \| 0.866 \| \| 0.573 \| \| 0.791 \| \| 0.698 \| \| 0.526 \| \| 0.567 \| | \| ** \| \| --- \| \| * \| \|  \| \|  \| \|  \| \|  \| \| * \| \|  \| |
| TX-3 | X.2  3.2  4.7  5.13  6.18  8.6  9.1  9.24 | \| 25 \| \| --- \| \| 25 \| \| 25 \| \| 25 \| \| 25 \| \| 23 \| \| 21 \| \| 25 \| | 14  9  12  3  5  4  7  4 | 1  1 | \| 0.720 \| \| --- \| \| 0.600 \| \| 0.480 \| \| 0.600 \| \| 0.880 \| \| 0.739 \| \| 0.333 \| \| 0.480 \| | \| 0.872 \| \| --- \| \| 0.664 \| \| 0.894 \| \| 0.496 \| \| 0.731 \| \| 0.643 \| \| 0.557 \| \| 0.670 \| | \|  \| \| --- \| \|  \| \| ** \| \|  \| \|  \| \|  \| \| * \| \|  \| |
| VA-1 | X.2  3.2  4.7  5.13  6.18  8.6  9.1  9.24 | \| 24 \| \| --- \| \| 23 \| \| 24 \| \| 24 \| \| 24 \| \| 24 \| \| 21 \| \| 24 \| | 12  8  12  4  4  4  2  4 |  | \| 0.792 \| \| --- \| \| 0.261 \| \| 0.500 \| \| 0.542 \| \| 0.833 \| \| 0.542 \| \| 0.095 \| \| 0.750 \| | \| 0.901 \| \| --- \| \| 0.737 \| \| 0.840 \| \| 0.530 \| \| 0.650 \| \| 0.608 \| \| 0.372 \| \| 0.602 \| | \|  \| \| --- \| \| ** \| \| ** \| \|  \| \|  \| \|  \| \| * \| \|  \| |


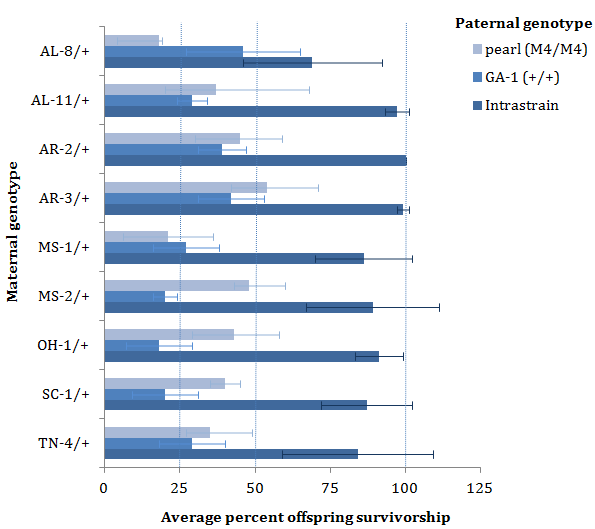


**Figure S1. M^4^ could not rescue mortality of individuals PCR-genotyped as M^1^-homozygous.** The results shown represent several crosses from sample locations determined to be M^1^-fixed using our PCR marker. Females used in these crosses are the result of wild beetles crossed to non-*Medea* strain GA-1. For example, AL-8 individuals typically genotyped as M^1^ homozygous, thus "AL-8/+" females are presumed to be heterozygous for M^1^. Averages and standard deviations of 3-5 crosses per genotype pair are shown. In each case, M^4^ was not sufficient to rescue mortality in crosses involving a female bearing a PCR-genotyped M^1^ allele.

Survivorship expectations are:

100% when *Medea's* mortality is rescued (or when the mother is non-*Medea*)

50% when an M^1^-heterozygous female is crossed to a non-*Medea* male

25% when an M^1^, M^4^ doubly-heterozygous female is crossed to a non-*Medea* male

**(a)**


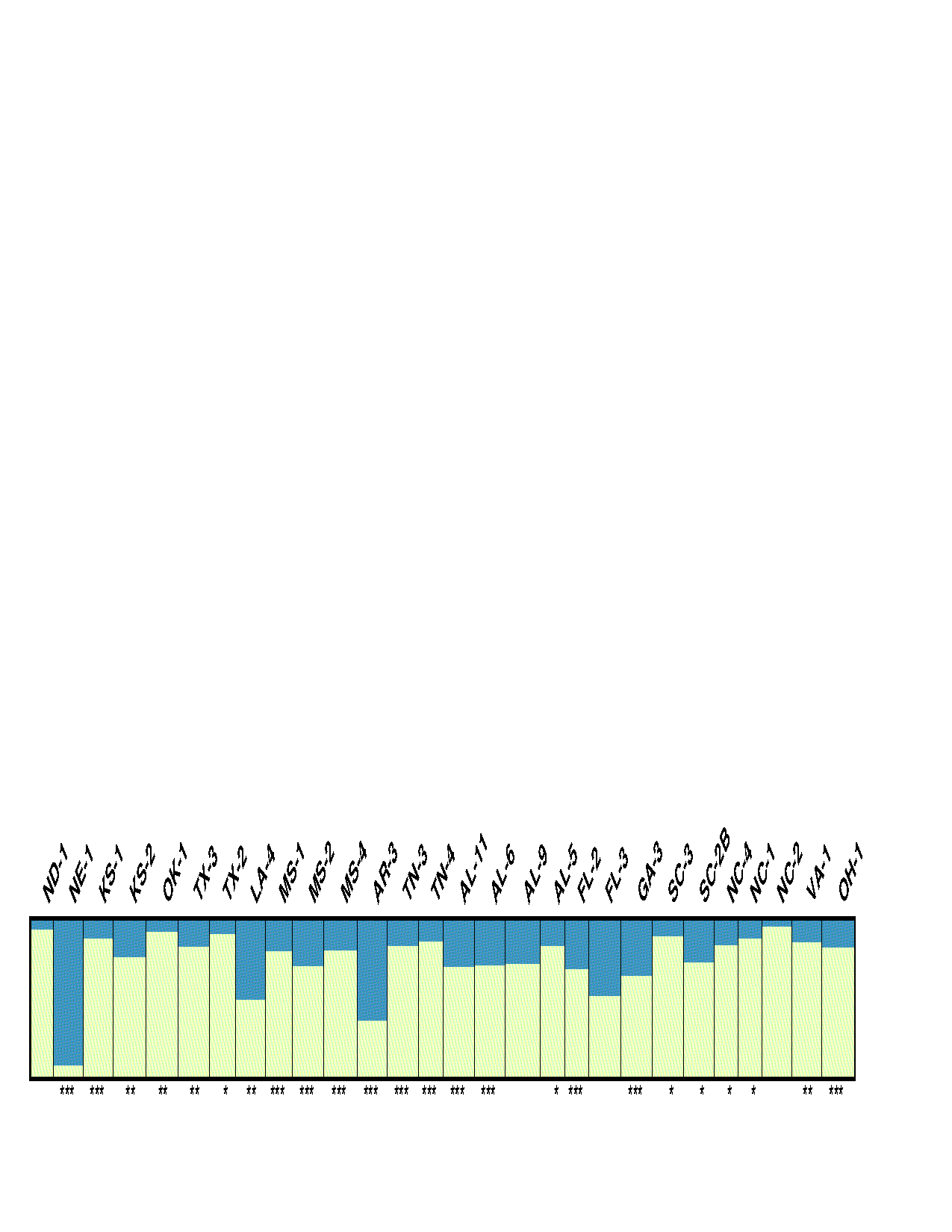


**(b)**


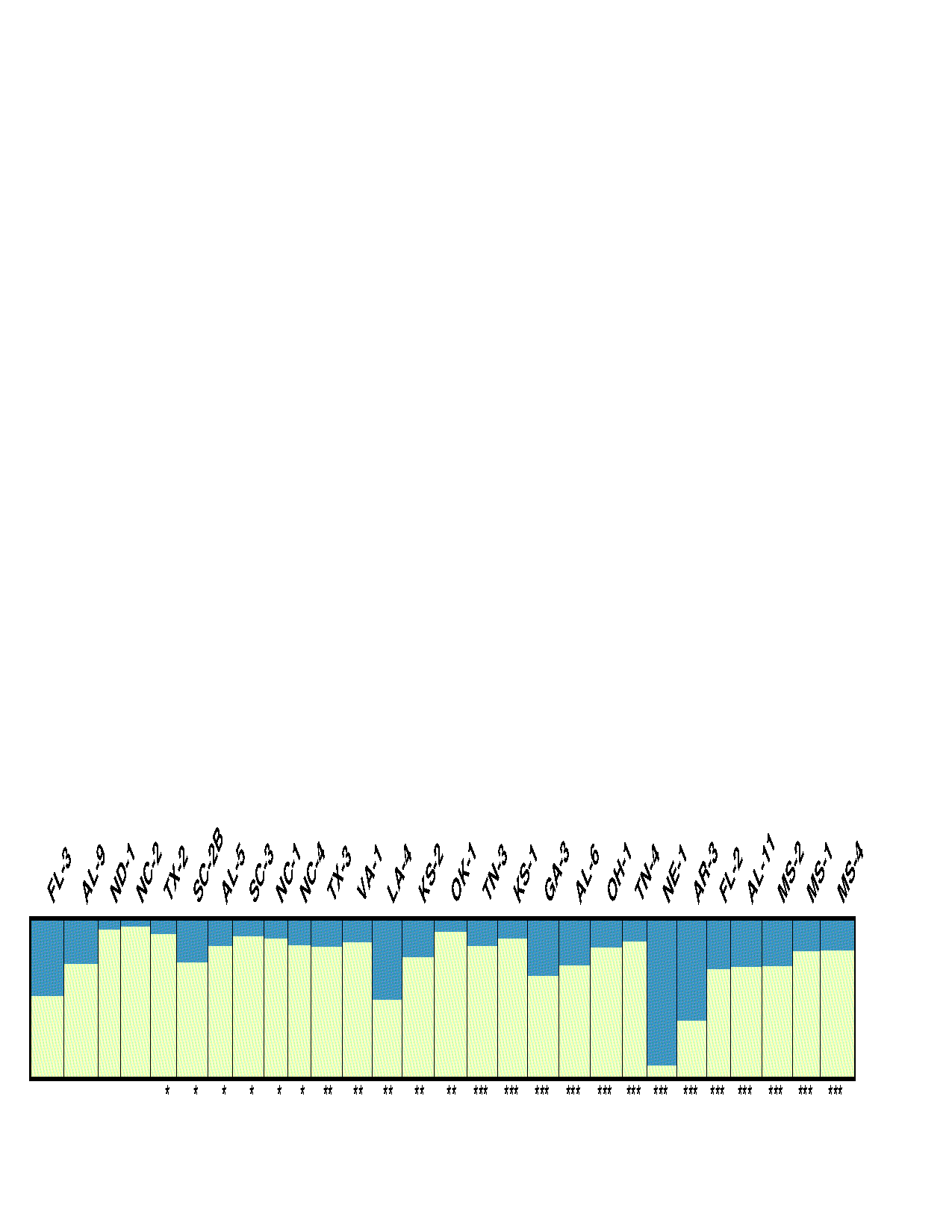


**(c)**


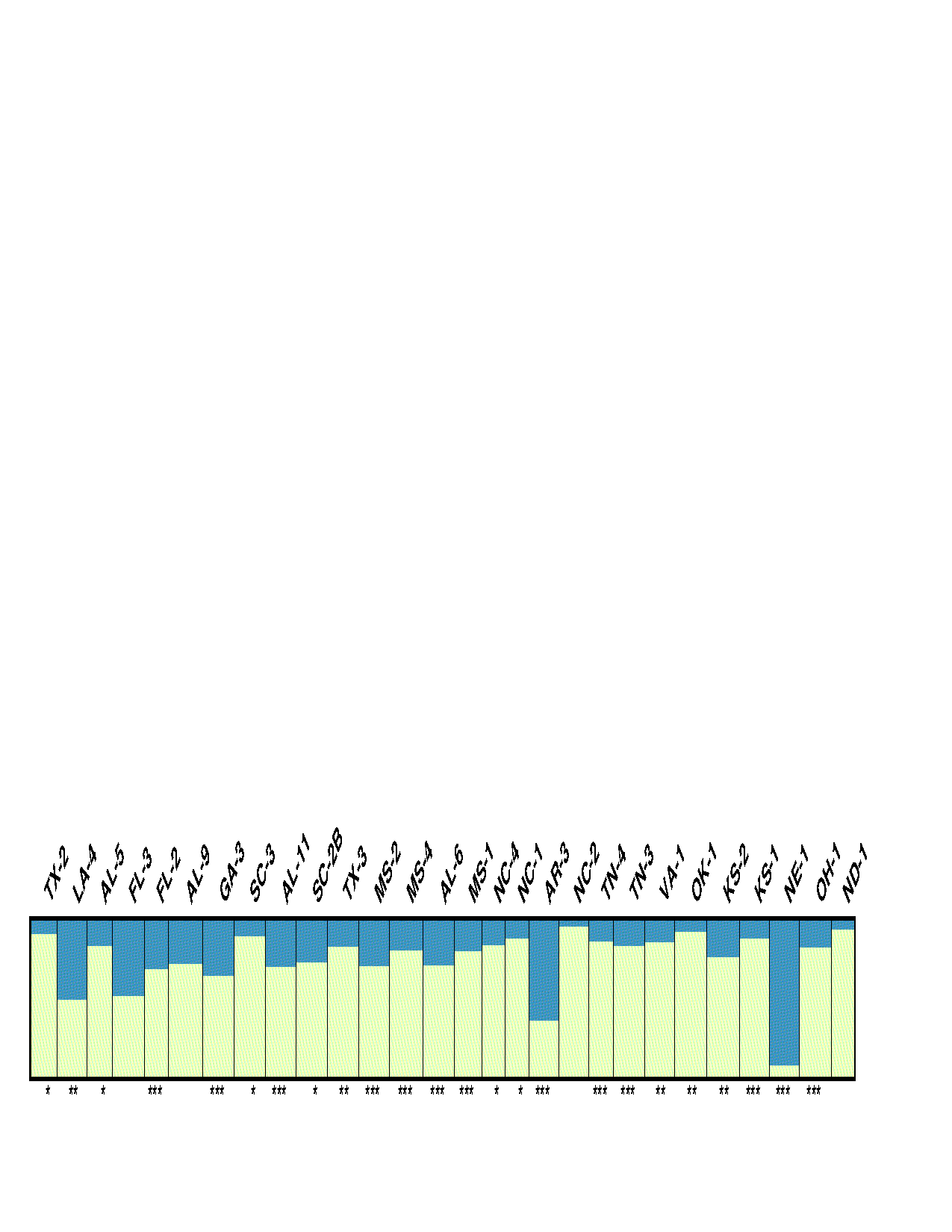


**Figure S2. STRUCTURE output at K = 2 shows no clear connection between genetic differentiation and M^1^.** Assignments of each individual tested to the two predicted clusters are shown. Above the figure are the states of origin for each sample location arranged **(a)** geographically, **(b)** by sample site M^1^ allele frequency and **(c)** by latitude. Below the figure are the general M^1^ genotypes for each location (no asterisks indicates **wild-type**, ***** indicates a **low** M^1^ genotype frequency between 0.04 and 0.19, ****** is an **intermediate** M^1^ genotype frequency between 0.39 and 0.5, ******* is a **HIGH** genotype frequency of at least 0.75, and typically indicates fixed samples). In (c), all sites to the right of AL-9 are above 33°N latitude.

**Table S6. Structure analysis results.** For each assessed value of K, the average and standard deviation of the likelihood of the data given that value of K are provided, as well as each value of ΔK.

| **K** | **Mean ln Pr(X\|K)** | **SD of ln Pr(X\|K)** | **ΔK** |
| --- | --- | --- | --- |
| 1 | -16162.08 | 0.11 |  |
| 2 | -16253.52 | 90.23 | **5.06** |
| 3 | -15888.54 | 115.90 | 0.68 |
| 4 | -15601.90 | 88.85 | 2.32 |
| 5 | -15521.54 | 97.66 | 0.24 |
| 6 | -15418.00 | 137.67 | 0.30 |
| 7 | -15273.50 | 99.39 | 3.23 |
| 8 | -15450.10 | 561.29 | 0.96 |
| 9 | -15089.02 | 108.49 | 3.33 |
| 10 | -15088.80 | 132.05 | 1.69 |
| 11 | -16162.08 | 0.11 | 1.35 |
| 12 | -16253.52 | 90.23 | 0.80 |
| 13 | -15888.54 | 115.91 | 0.59 |
| 14 | -15601.90 | 88.85 | 2.84 |
| 15 | -15521.54 | 97.66 |  |

**Table S7. Global per-locus estimates of differentiation.** Shown are per-locus values of F_ST_ (both uncorrected and ENA-corrected) and R_ST_, as well as averages across all loci.

| **Locus** | **Uncorrected F_ST_** | **ENA-Corrected F_ST_** | **R_ST_** |
| --- | --- | --- | --- |
| **X.2** | 0.03627 | 0.03654 | 0.0613 |
| **3.2** | 0.08579 | 0.07064 | 0.0953 |
| **4.7** | 0.07815 | 0.06707 | 0.0729 |
| **5.13** | 0.05678 | 0.05719 | 0.0411 |
| **6.18** | 0.05357 | 0.05383 | 0.0927 |
| **8.6** | 0.05891 | 0.05922 | 0.0836 |
| **9.1** | 0.11464 | 0.10827 | 0.1127 |
| **9.24** | 0.11045 | 0.10734 | 0.1656 |
| **All loci** | 0.07163 | 0.06695 | 0.0932 |

**Figure S3. A weak positive association between genetic distance and geographic distance was found using microsatellite loci and beetles sampled across the United States.** Pairwise F_ST_/(1-F_ST_) is plotted against the log of the distance (in kilometers) between sample site pairs. Both the uncorrected and ENA-corrected F_ST_ values were analyzed.


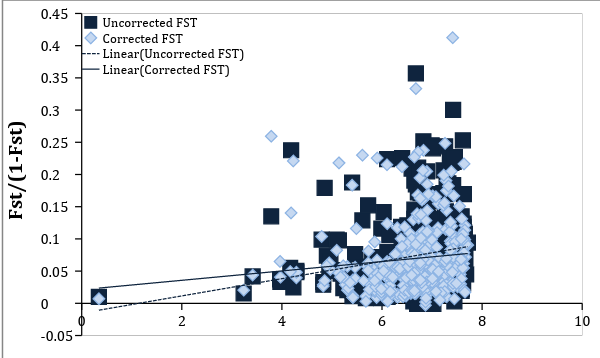

Supplement: Supplementary file 1 [file ECE3-9-14407-s001.docx]
